# Biopsychosocial Predictors of Quality of Life in Paediatric Patients With Sickle Cell Disease

**DOI:** 10.3389/fpsyg.2021.681137

**Published:** 2021-09-14

**Authors:** Anna M. Hood, Melanie Kölbel, Hanne Stotesbury, Jamie Kawadler, April Slee, Baba Inusa, Maria Pelidis, Jo Howard, Subarna Chakravorty, Sue Height, Moji Awogbade, Fenella J. Kirkham, Christina Liossi

**Affiliations:** ^1^Developmental Neurosciences Unit and Biomedical Research Centre, University College London Great Ormond Street Institute of Child Health, London, United Kingdom; ^2^Department of Primary Care and Population Health, University College London, London, United Kingdom; ^3^Department of Paediatric Haematology, Evelina London Children's Hospital, Guy's and St Thomas' NHS Trust, London, United Kingdom; ^4^Department of Haematological Medicine, King's College London, London, United Kingdom; ^5^Department of Haematology, Guy's and St Thomas' NHS Foundation Trust, London, United Kingdom; ^6^Paediatric Haematology, King's College Hospital NHS Trust, London, United Kingdom; ^7^Department of Haematological Medicine, King's College Hospital NHS Trust, London, United Kingdom; ^8^Department of Clinical Haematology, University College London Hospitals NHS Foundation Trust, London, United Kingdom; ^9^Department of Child Health, University Hospital Southampton NHS Foundation Trust, Southampton, United Kingdom; ^10^Clinical and Experimental Sciences, University of Southampton, Southampton, United Kingdom; ^11^Department of Psychology, University of Southampton, Southampton, United Kingdom; ^12^Paediatric Psychology, Great Ormond Hospital for Children NHS Foundation Trust, London, United Kingdom

**Keywords:** executive function, pain burden, sleep, emergency department visit, coping

## Abstract

Sickle cell disease (SCD) refers to a group of inherited blood disorders with considerable morbidity that causes severe pain, reduces life expectancy, and requires significant self-management. Acute painful episodes are the hallmark of SCD, but persistent daily pain is also highly prevalent in this population. Characterising the impact and experience of SCD-related morbidity (i.e., sleep disruption, frequent emergency department visits, cognitive dysfunction) on health-related quality of life (HRQOL) requires multiple assessment methods to best capture the underlying mechanisms. To gain a greater understanding of the effect of common symptom categories on HRQOL and to determine potential pain coping targets, the present study investigated whether demographic, socioeconomic, sleepiness, pain burden, frequency of emergency department (ED) visits, and cognition predicted HRQOL in a paediatric sample of patients with SCD. Our study was a secondary analysis of baseline assessment data of children with SCD aged 8–15 years (*n* = 30) in the Prevention of Morbidity in Sickle Cell Anaemia Phase 2b (POMSb2) randomised controlled clinical trial of auto-adjusting continuous positive airways pressure. Patients completed cognitive testing (IQ, Processing Speed Index, Delis-Kaplan Executive Function Scale (DKEFS) Tower, Conner's Continuous Performance Test), sleepiness (Epworth Sleepiness Scale), and HRQOL (PedsQL Sickle Cell Module) at baseline. Patients reported pain burden (Sickle Cell Pain Burden Inventory-Youth) each month over 8 visits. Caregivers provided demographic information and reported their child's executive function (Behavioural Rating Inventory of Executive Function) at baseline. Data from our analysis demonstrated that demographic factors (i.e., age, gender, level of neighbourhood deprivation) and treatment variables (i.e., hydroxyurea use) did not independently predict HRQOL, and laboratory values (i.e., haemoglobin, haematocrit, mean oxygen saturation) were not significantly correlated with HRQOL (*ps* > 0.05). However, sleepiness, pain burden, ED visits, and executive dysfunction independently predicted HRQOL (*R*^2^ = 0.66) with large effects (η^2^ = 0.16 to 0.32). These findings identify specific, measurable symptom categories that may serve as targets to improve HRQOL that are responsive to change. This knowledge will be useful for multimodal interventions for paediatric patients with SCD that include sleep management, pain coping strategies, and executive function training.

## Introduction

Sickle cell disease (SCD) is a group of genetic disorders that affect the structure and oxygen-carrying capacity of haemoglobin in red blood cells (Rees et al., 2010). SCD can impact multiple systems, with symptoms including an increased risk of infection, cognitive complications, and damage to the organs and bones (Redding-Lallinger and Knoll, 2006; Booth et al., 2010; DeBaun and Kirkham, 2016). Globally, it is estimated that 300,000 infants with SCD are born each year, with the majority born in Sub-Saharan Africa (Piel et al., 2013; Kato et al., 2018). Patients with SCD experience recurrent acute painful episodes with persistent pain between episodes (Dampier et al., 2002; Smith et al., 2008). The occurrence, location, severity, and duration of pain episodes and persistent pain can vary substantially in an individual patient tending to worsen with age (Ballas, 2020). These types of pain can sometimes have precipitating factors or objective signs specific to the individual patient (e.g., infection, fever, obstructive sleep apnoea, dehydration) and changes in the environment associated with increased admissions for pain in the population (e.g., increased wind speed, rainfall) (Piel et al., 2017). Importantly, unpredictable fluctuations can also lead to uncertainty (Gil et al., 2003). As such, patients with SCD have a unique pain profile (Yawn et al., 2014) that can negatively impact quality of life (Sil et al., 2016). Patients with SCD generally experience poorer quality of life than national norms and other patients with chronic conditions (i.e., cystic fibrosis, asthma, haemodialysis patients) with worsening quality of life as pain increases (McClish et al., 2005).

For patients with SCD, coping with pain and the associated complex treatment regimen (e.g., frequent hospital contacts) is often associated with comorbid psychological symptoms (e.g., depression, anxiety) (Benton et al., 2007; Graves et al., 2016), impaired family functioning (Oliver-Carpenter et al., 2011), chronic fatigue (Ameringer et al., 2014), and sleep disturbances (Kaleyias et al., 2008), all of which may be exacerbated by potentially challenging socioeconomic and environmental factors. Given the aetiology of pain in paediatric patients with SCD, assessing morbidity requires an integrative biopsychosocial approach to evaluating, formulating, and managing pain. To move beyond just measuring pain intensity, information on a wide range of relevant dimensions (i.e., biological, psychological, and sociocultural factors) using multiple assessment methods is needed to capture the patients' specific experiences and understand the causes, contributors, and effects of pain (Liossi and Howard, 2016).

Evaluation and treatment utilising the biopsychosocial approach enables patients to actively manage their condition and improve coping resources. Coping refers to the dynamic processes (behavioural and psychological) that people employ to manage or reduce stress when faced with adverse experiences, including pain or disease-related distress (Skinner and Zimmer-Gembeck, 2007). Pain coping is often categorised into strategies defined as adaptive (e.g., active activities like exercise, meditation, listening to music) and maladaptive (e.g., catastrophising). A similar dichotomy has been posited between “approach vs. avoidant” coping strategies, which describe engaging with or avoiding pain, respectively (Van Damme et al., 2008) and “problem-focused vs. emotion-focused” strategies, which describe efforts to control and emotionally manage pain, respectively (Lazarus and Folkman, 1984).

Consideration of potential cultural factors may account for some variation in pain coping strategies used by patients with SCD. For example, Oliver-Carpenter et al. (2011) found that children and adolescents with SCD used passive (e.g., resting, drinking fluids) rather than positive coping attempts (e.g., active coping strategies) most often. These mostly avoidant or passive pain coping strategies are related to more emergency department visits, higher pain intensity, and lower activity levels (Gil et al., 1991; Anie et al., 2002). Additionally, children with SCD have been shown to experience pain that affects sleep patterns and how they cope with pain (e.g., behavioural distraction or catastrophising) (Graves and Jacob, 2014). However, it has also been shown that patients with SCD with and without abnormal sensory patterns (e.g., enhanced pain sensitivity) frequently use positive approaches (e.g., seeking social support) for coping with pain (Hyacinth et al., 2020).

Distinctions between different types of coping strategies are not as clear cut as they may seem; however, as adaptiveness can vary with context, culture, and for the individual. Further, both types of strategies can be helpful for some patients in some settings. For example, recent studies have demonstrated that spiritual coping strategies influence health outcomes depending on whether they are more positive (e.g., seeking comfort and strength) or more negative (e.g., spiritual doubts) (Reynolds et al., 2013). Because Black individuals engage in more spiritual coping strategies (e.g., prayer) than White individuals and because most commonly used measures of coping conceptualise prayer as a passive/avoidant strategy (Meints et al., 2016) the potential adaptiveness of spirituality can be obscured.

Psychosocial interventions that empower patients with chronic conditions to learn coping skills can improve their ability to manage and reduce persistent pain (Somers et al., 2018). Addressing the biopsychosocial factors related to persistent pain is particularly important for paediatric patients with SCD as they often lack the necessary skills and confidence to effectively manage their disease (Abel et al., 2015; Stollon et al., 2015). Study endpoints for psychosocial interventions for paediatric patients with SCD often aim to improve health-related quality of life (HRQOL) (Anderson et al., 2018; Adegbolagun et al., 2020). HRQOL is a multidimensional construct that includes health risks, conditions, functional status, physical and mental health, and social support (Ferrans et al., 2005). Specific to patients with SCD, better HRQOL is related to improved treatment, psychosocial and psychological outcomes and fewer hospital contacts (Thornburg et al., 2011; Beverung et al., 2015a; Sil et al., 2016; Hood et al., 2020b). In relation to pain coping, mediation analyses have shown that the relationship between pain and physical HRQOL was mediated by emotion-focused avoidance pain coping. Specifically, emotion-focused avoidance coping was related to worse pain and, in turn, decreased physical HRQOL (Lim et al., 2019). Relatedly, negative thinking mediated the role between pain and internalising psychological symptoms (Barakat et al., 2008). Finally, in a study of adults with SCD, affective coping strategies (e.g., anger and fear self-statement, isolation) significantly predicted poor HRQOL (Anie et al., 2002).

In addition to overall poorer HRQOL (Panepinto et al., 2013), cognitive challenges, particularly in the domains of processing speed (Stotesbury et al., 2018), attention (Daly et al., 2012), and executive function (Hood et al., 2019) are frequently observed in patients with SCD and have also been suggested as efficacious endpoints for clinical trials in this population (Farrell et al., 2019). The aetiology of cognitive difficulties in patients with SCD are multi-faceted (Prussien et al., 2020). There is some evidence for relationships between cognitive dysfunction and pain (Connolly et al., 2019), sleep (Marshall et al., 2009), pain coping (Ludwig et al., 2018), and HRQOL (Allen et al., 2017; Hardy et al., 2018). Two randomised control trials (RCT) have begun to utilise smartphone technology to improve pain outcomes (e.g., reduce emergency department visits) by targeting sleep hygiene techniques, pain tracking, and self-management (Schatz et al., 2015; Palermo et al., 2018). Nevertheless, more work is needed to better understand the relative impact of these symptom categories on HRQOL, along with the potential impact of cognitive difficulties. Moreover, studies have often considered one or two symptoms or aspects of SCD-related morbidity rather than considering multiple potential biopsychosocial factors in one paediatric cohort.

Given that utilisation of pain coping strategies improve the management of these symptom categories and complications (i.e., sleep, cognition, ED visits) and that pain coping interventions often aim to improve HRQOL, the purpose of our secondary analysis is to assess whether biopsychosocial factors that are frequent pain coping targets predict HRQOL. Gaining a deeper understanding of these factors will allow for targeted interventions for paediatric patients with SCD. Through the inclusion of multiple assessment tools that are often used when examining the effects of pain coping, we collected baseline data in the paediatric arm of the Prevention of Morbidity in Sickle Cell Disease Phase 2b (POMS2b) trial (Howard et al., 2018), we aimed to determine whether demographic, socioeconomic, sleep, pain burden, emergency department visits, and cognition predicted HRQOL in patients with SCD. We focused on cognitive data in domains where patients with SCD experience the most profound challenges (i.e., executive function, attention, and processing speed). The present study aims to understand what symptom categories and complications have the most significant effect on HRQOL for paediatric patients with SCD and provide knowledge for future multimodal interventions.

## Materials and Methods

### Procedures

**Figure 1:**
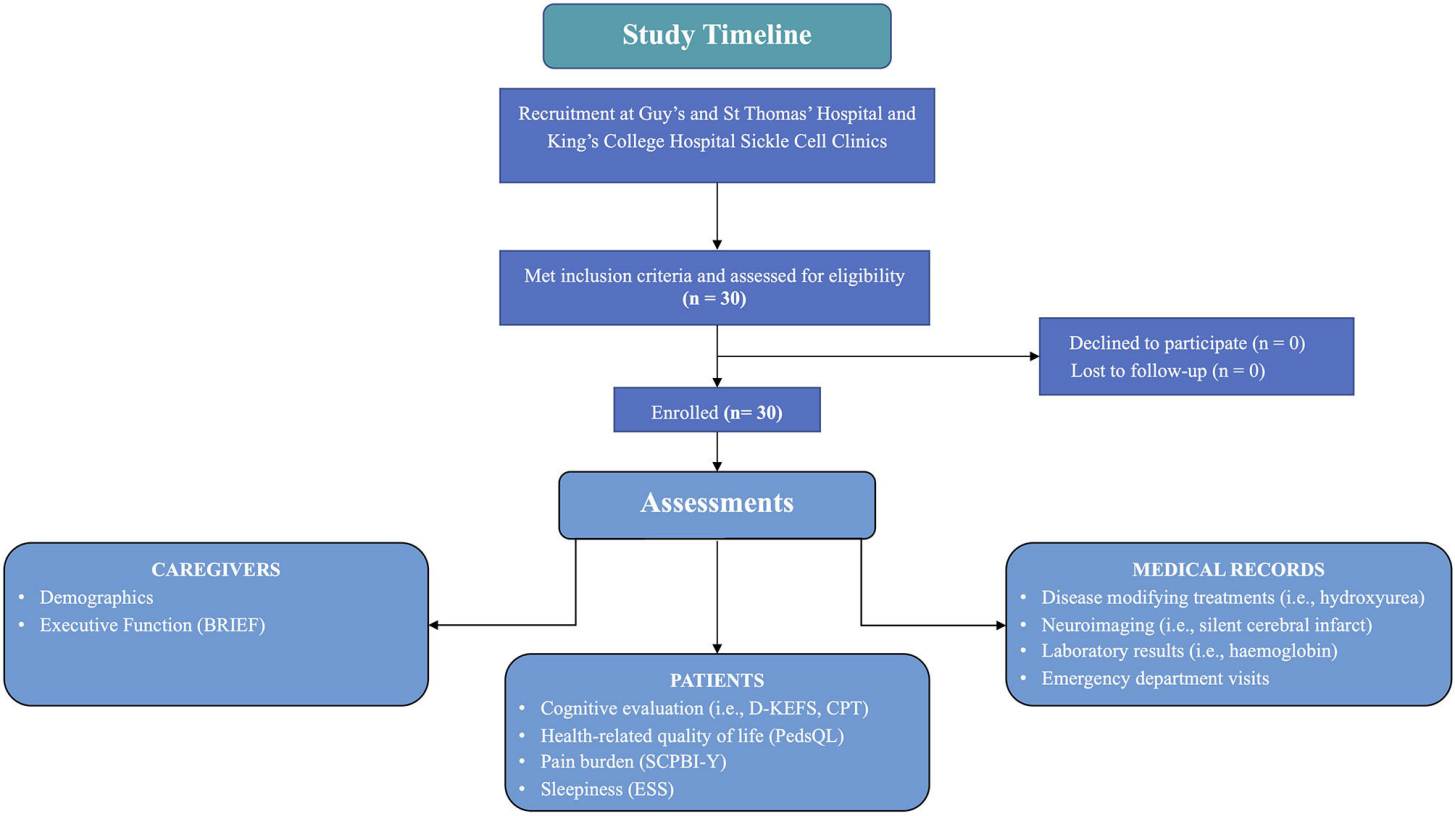
Study timeline. SCPBI-Y, Sickle Cell Pain Burden Interview-Youth; Conners' Continuous Performance Test-Third Edition, CPT-3; PedsQL, Paediatric Quality of Life Inventory HRQOL; DKEFS, Delis-Kaplan Executive Function System; BRIEF GEC, Behavioural Rating Inventory of Executive Function Global Executive Composite.

This study reports data from a *post hoc* secondary analysis of paediatric data (*n* = 30) collected before randomisation for the single-blind, randomised, controlled phase II trial (POMS2b). Baseline included all visits (i.e., cognitive testing and completion of caregiver and self-report) up to and including the day of randomisation (see Figure 1 for a study timeline). The POMS2b trial compared 6-months of overnight auto-adjusting continuous positive airways pressure (APAP) with standard care (*n* = 30) to standard care alone (*n* = 30) in paediatric patients (children ≤16 years of age) and adults with sickle cell anaemia (HbSS genotype). The trial aimed to determine whether the intervention was safe and whether there were posttreatment improvements in cognition and brain structure. Descriptions of the study design and findings have been published elsewhere (Howard et al., 2018, 2019; Slee et al., 2019).

Participants were recruited through sickle cell clinics at the Guy's and St Thomas' Hospital and King's College Hospital in the United Kingdom (UK), serving ~460 and 480 patients with SCD, respectively. Trained PhD and MSc neuropsychology students administered cognitive testing in a quiet room at University College London. Inclusion criteria for the paediatric arm of the POMS2b trial were being 8 to 15.99 years of age, having the ability to speak and understand English, a diagnosis of sickle cell anaemia (HbSS genotype). Exclusion criteria included mean overnight saturation of <90% for >30% of the night (i.e., meeting current criteria for overnight oxygen therapy), current or prior experience with overnight respiratory support, respiratory or decompensated cardiac failure, hospital admission for acute sickle complications within the past 1 month and more than 6 hospital admissions in the last 12 months, receiving chronic transfusions, or contraindications to APAP therapy or MRI. The study received ethical approval from the NRES Committee East of England—Cambridge South (14/EE/0163).

### Measures

#### Demographics and Clinical Characteristics

Caregivers reported on child demographics. We obtained medical information through electronic medical chart review, including identifying if patients received common disease-modifying SCD treatments (i.e., hydroxyurea). Active medical problems documented in electronic medical records were reviewed to obtain neurological complications such as stroke history, abnormal magnetic resonance imaging (MRI; indicative of structural brain abnormalities), medical laboratory results (i.e., mean overnight oxygen saturation, haemoglobin, haematocrit), and the number of emergency department (ED) visits within the past year.

The English Indices of Deprivation are widely used open-source data that provide official government measures of relative deprivation in England (Ministry of Housing, Communities & Local Government, 2019). The Index of Multiple Deprivation (IMD) defines deprivation as encompassing seven different domains which are weighted as follows (i.e., income [22.5%], employment [22.5%], education [13.5%], health [13.5%], crime [9.3%], housing/services barriers [9.3%], and living environment [9.3%]), and is obtained through the patient's home postcode. These domains each have multiple components. For example, the housing and services barriers domain includes household overcrowding, homelessness etc. Neighbourhoods are ranked on a relative rather than absolute scale according to their level of deprivation relative to that of other areas. Zones are grouped into 5 bands (quintiles), each containing 20% of the data zone, with “1 = most deprived, 5 = least deprived.”

#### Paediatric Quality of Life Inventory Sickle Cell Disease Module (PedsQL)

Participants completed the PedsQL (Panepinto et al., 2013) at baseline, which is a 43-item multidimensional rating scale that assesses HRQOL in children with SCD. Caregivers rated how much of a problem an issue had been for their child on a 5-point scale of “Never” to “Almost Always.” Responses were reverse-scored and linearly transformed to a 0–100 scale (0 = 100, 1 = 75, 2 = 50, 3 = 25, 4 = 0). Total scores were then computed as the sum of the items divided by the number of items answered. Total scores and clinical classifications (81–100 = high levels of HRQOL, 61–80 = intermediate levels HRQOL, and 0–60 = poor HRQOL related to pain) were used in analyses (Beverung et al., 2015b).

#### Sickle Cell Pain Burden Interview-Youth (SCPBI-Y)

We assessed pain burden through the SCPBI-Y. Participants completed the SCPBI-Y at baseline, which is a 7-item multidimensional interview assessing the impact of pain on functional ability, sleep, school, and mood over the past month. Each item is rated on a 5-point Likert scale from “none = 0” to “every = 4.” Scores range from 0 = no pain burden to 28 = severe pain burden. Scores from 8 months of visits were averaged and used in the present study to improve the reliability of the estimate and to best capture the experience of chronic or persistent pain (i.e., 12 weeks or more).

#### Epworth Sleepiness Scale (ESS)

Patients completed the Epworth Sleepiness Scale (ESS) (Johns, 1991) at baseline, which is an 8-item questionnaire that assesses the chance of dozing off or falling asleep while engaged in eight different activities. Responses are rated on a 4-point scale of 0 = “would never doze” to 3 = “high chance of dozing.” The ESS total score is the sum of all items and can range from 0 to 24. The higher the ESS score, the higher the child's daytime sleepiness.

#### Delis-Kaplan Executive Function System (D-KEFS) Tower Test

Participants completed the D-KEFS (Delis et al., 2001) at baseline. The D-KEFS comprises 9 tests with normed scores for children aged 8 to 18 years that represent domains of executive function. The Tower subtest was used in the present study and primarily assesses planning and problem-solving, as well as learning rules, inhibiting impulsivity, and maintaining instructional sets. Participants have to build a series of progressively more difficult towers using disks of various sizes in as few moves as possible across three pegs, following pre-specified rules and within a time limit. The Tower test is discontinued after three consecutive failures. Tower raw scores were converted to standardised scores (M= 10, SD = 3) and examined using the summary score (i.e., Total Achievement Score), which is the sum of achievement points earned for all completed items. A higher total achievement score represents better overall executive function.

#### The Conners' Continuous Performance Test-Third Edition

Participants completed the Conners' Continuous Performance Test-Third Edition (CPT-3) (Conners, 2000) at baseline, which is a 14 min computerised test of sustained attention and impulsivity where letters are displayed on a computer monitor one at a time. Participants observe the stimuli and press the space bar as quickly as possible after each letter, except for the letter X. Outcome measures used in the present study are omission errors which represent the number of times the target was presented, but the participant did not respond (i.e., distractibility) and commission errors which represent the number of times the participant responded but no target was present (i.e., impulsivity). Higher T-scores (> 65) indicate clinical concern (i.e., poorer sustained attention).

#### Behavioural Rating Inventory of Executive Function

Caregivers completed the 86-item Behavioural Rating Inventory of Executive Function (BRIEF) at baseline. The BRIEF assesses EF behaviour in the school and home environments (Gioia et al., 2000). The BRIEF scales include: Inhibit, Shift, Emotional Control, Self-Monitor, Initiate, Working Memory, Planning/Organisation, Task Monitoring, and Organisation of Materials. The Global Executive Composite (GEC), which incorporates scores from all scales, was used in our analyses. Higher T-scores (>65) indicate clinical concern (i.e., greater executive dysfunction).

#### Wechsler Tests

Participants completed tests at baseline. The Wechsler Abbreviated Scale of Intelligence (WASI) (Wechsler, 1999) which was used to estimate the intelligence quotient (IQ; Mean = 100, SD = 15) and the Wechsler Intelligence Scale for Children Fourth-Edition (WISC-IV) (Wechsler, 2003) was used to measure the Processing Speed Index (PSI) using the scores from the Symbol Search, Coding or Cancellation (substituted when required) subtests.

### Statistical Analyses

The R statistical package was used to conduct analyses (Team, 2016). Descriptive statistics and summary scores with percentages described the overall sample. Visual inspection of our data using histograms along with skew and kurtosis analyses indicated that the number of ED visits within the past year and pain burden were both positively skewed (i.e., mostly lower values). Therefore, these two variables were log-transformed. One-sample *t*-tests assessed differences in sample cognitive scores and the normative population mean. Bivariate Pearson correlations assessed linear relationships between continuous predictors of interest. Two hierarchical linear regression analyses were conducted assessing predictors of HRQOL. To test the most parsimonious model, we only included potential predictors in models if correlations with HRQOL were above *r* = 0.2. To increase the power of our models, demographic [i.e., age, gender, deprivation (IMD)] and medical variables (i.e., receiving hydroxyurea) were only included in linear regression models if they were significant predictors of HRQOL (*ps* < 0.05).

To reduce multicollinearity, we conducted two separate regression models assessing whether psychosocial measures and tests of executive function (i.e., BRIEF GEC) and attention (i.e., CPT commissions errors) predicted HRQOL over and above the influence of symptom categories (i.e., sleepiness, pain burden, ED visits). We considered *p* < 0.05 two-tailed as statistically significant. Partial eta squared ($\eta_{\text{p}}^{2}$) was the measure of effect size used for the linear regression analyses. η^2^ = 0.01, 0.06, and 0.14 represented small, medium, and large effect sizes, respectively (Cohen, 1988). Bias and corrected 95% confidence intervals were used as interval estimates to adjust for possible bias and skewness.

## Results

### Participant Characteristics

Baseline demographic characteristics are displayed in Table 1. The mean age of participants was 12.3 years (40% female). Most participants lived in the most deprived regions in the UK. Nearly half had silent cerebral infarction on MRI. Less than a quarter had a pre-existing diagnosis of asthma. The number of ED visits within the past year was low, with 86% of participants having zero visits. Just over half of the sample were receiving hydroxyurea. Cronbach's alpha's for patient-reported outcome measures ranged from 0.66 to 0.98, demonstrating adequate to excellent internal consistency (see Table 2).

**Table 1:** Characteristics of POMS2b paediatric patients at baseline before randomisation.

**Characteristics**	***N* = 30**
**Age**	
Mean (SD)	12.3 (2.1)
Range	8–15
	***N*** **(%)**
**Sex**	
Female	12 (40%)
Male	18 (60%)
**IMD quintile (1** **=** **most deprived)**	
1	13 (43.3%)
2	15 (50%)
3	2 (6.7%)
4	0 (0%)
5	0 (0%)
**Genotype**	
HbSS	30 (100%)
**Silent cerebral infarct on MRI**	
No	16 (53.3%)
Yes	14 (46.7%)
**Prescribed hydroxyurea**	
No	14 (46.7%)
Yes	16 (53.3%)
**Diagnosis of asthma**	
No	23 (76.7%)
Yes	7 (23.3%)
	**Mean (SD)**
Mean oxygen saturation (%)	95.8 (2.8)
Haemoglobin level (g/l)	89.3 (11.9)
Haematocrit level (volume %)	27.2 (3.9)
	**Median (range)**
Number of ED visits in the past year	0 (0–2)

*SD, Standard deviation; IMD, Index of Multiple Deprivation; MRI, Magnetic Resonance Imaging; ED, Emergency department*.

**Table 2:** Patient-reported outcomes for POMS2b paediatric participants at baseline before randomisation.

**Measures**	**α**	***N* = 30**
		**Mean (SD)**
Sickle Cell Pain Burden Interview-Youth	0.90	1.0 (1.7)
Range		0–28
Epworth Sleepiness Scale	0.66	4.6 (3.4)
Range		0–24
PedsQL Sickle Cell Disease Module	0.98	79.3 (21.3)
Range		0–100
**PedsQL clinical classifications [** ***N*** **(%)]**		
Poor		8 (26.7%)
Intermediate		3 (10%)
High		19 (63.3%)
BRIEF GEC T scores	0.93	51.5 (9.5)
Range		36–68
**BRIEF classifications**		
Within normal		25 (86.2%)
Clinically elevated		4 (13.79%)

*α, Cronbach's alpha; PedsQL, Paediatric Quality of Life Inventory; BRIEF GEC, Behavioural Rating Inventory of Executive Function Global Executive Composite. Higher T-scores are of greater clinical concern*.

**Figure 2:**
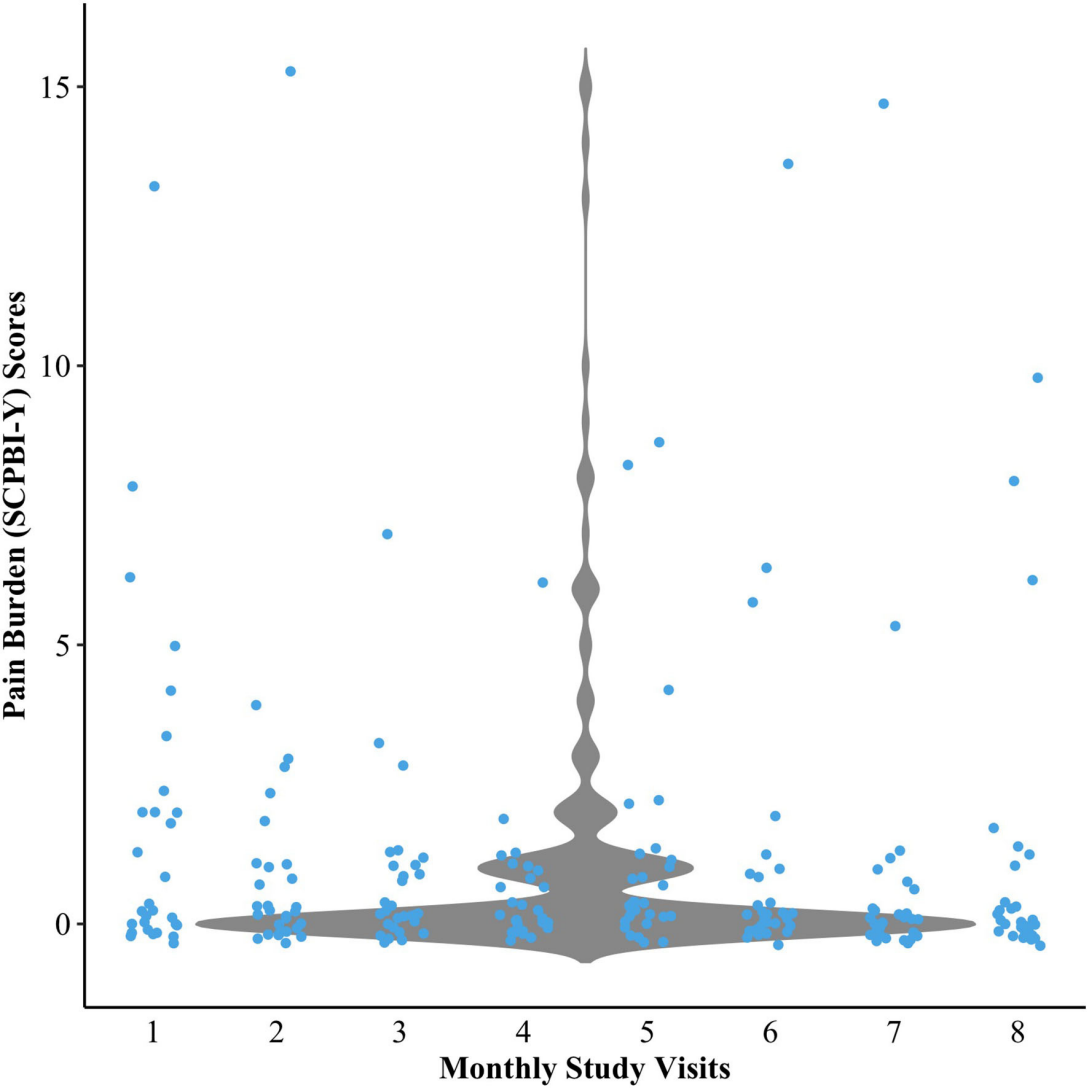
Violin plot demonstrating the variability in pain burden scores over 8 study visits. SCPBI-Y, Sickle Cell Pain Burden Interview-Youth.

Descriptive data from patient-reported outcomes indicated some variability in overall pain burden across study visits, but scores were generally low in the sample (see Figure 2). Sleepiness scores were generally low. Data further revealed that 37% of the sample had poor-to-intermediate HRQOL, and nearly 14% of the sample had clinically elevated BRIEF scores (indicative of executive dysfunction) (see Table 3). The cognitive scores of the overall sample indicate that IQ and CPT-3 omission errors were firmly in the average range and were not significantly lower than the normative population mean. In contrast, the PSI and DKEFS Tower total achievement scores were poorer than the population mean. CPT-3 commission errors were also significantly lower; however, only higher scores are indicative of impulsivity (see Table 3).

**Table 3:** Cognitive scores of POMS2b paediatric participants at baseline before randomisation.

**Cognitive scores**	**Sample *N* = 30**	**Normative population mean**	**Mean difference**	**Difference between the sample and the normative population mean**		
				**t**	***p***	**Cohen's *d***
	**Mean (SD)**					
Intelligence Quotient (IQ)	99.7 (13.2)	100 (15)	0.30	−0.14	0.89	0.03
Processing Speed Index (PSI)	93.9 (13.8)	100 (15)	6.1	2.38	**0.03**	0.44
DKEFS Tower achievement score	8.9 (2.0)	10 (3)	1.1	−3.00	**0.01**	0.55
CPT-3 omission errors	54.0 (14.7)	50 (10)	4	1.48	0.15	0.27
CPT-3 commission errors	46.4 (8.5)	50 (10)	3.6	2.24	**0.03**	0.42

*SD, Standard deviation; DKEFS, Delis-Kaplan Executive Function System; Conners' Continuous Performance Test-Third Edition, CPT-3; IQ and PSI, Standard Score; DKEFS, Scaled Score; CPT-3, T-score; Higher T-scores are of greater clinical concern. Significant differences are highlighted in bold*.

### Correlation Analyses

**Figure 3:**
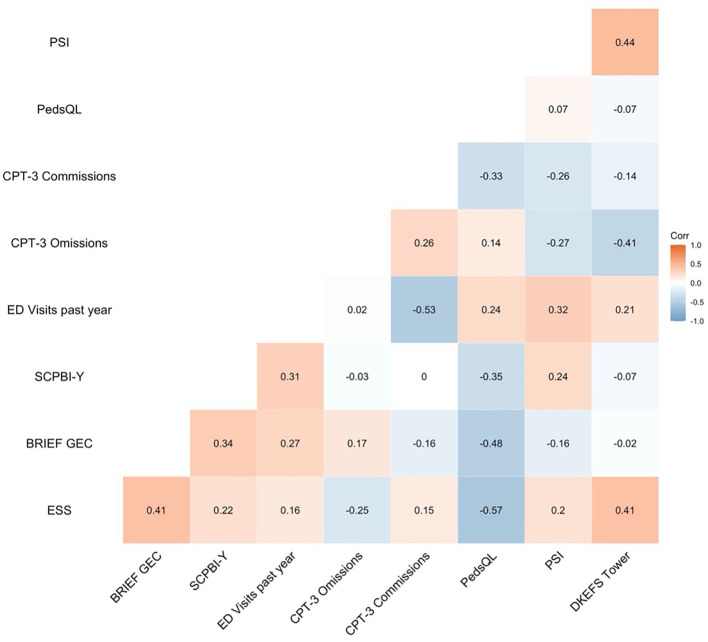
Bivariate correlations of patient-reported outcomes, emergency department visits, and cognition. SCPBI-Y, Sickle Cell Pain Burden Interview-Youth; Conners' Continuous Performance Test-Third Edition, CPT-3; ED, emergency department; PedsQL, Paediatric Quality of Life Inventory HRQOL; DKEFS, Delis-Kaplan Executive Function System; BRIEF GEC, Behavioural Rating Inventory of Executive Function Global Executive Composite.

Initial bivariate correlations demonstrated that HRQOL was not correlated with any baseline laboratory values (i.e., mean oxygen saturation, haemoglobin, or haematocrit) (*ps* > 0.05). Regarding patient-reported outcomes, HRQOL was significantly negatively correlated with the SCPBI-Y (*r* = −0.35) and the ESS (*r* = −0.57), indicating that those with poorer HQROL had higher pain burden and more sleepiness. In terms of cognition, HRQOL was significantly negatively correlated with CPT-3 commission errors (*r* = −0.33) and the BRIEF GEC (*r* = −0.48), indicating that those with poorer HQROL had more impulsivity and greater executive dysfunction (see Figure 3 for a correlation matrix of all potential predictors).

### Hierarchical Regression Analyses

Preliminary analyses indicated that no demographic [i.e., age, gender, deprivation (IMD)] or medical variables (i.e., hydroxyurea) were significant predictors of HRQOL (*ps* > 0.05), so they were not considered in subsequent linear regression models. As correlation analyses determined that the CPT-3 commission errors (impulsivity) and BRIEF GEC (executive dysfunction) were correlated with HRQOL, we assessed only these measures of cognition in regression analyses (a priori cut-off of *r* > 0.2).

Each regression model determined if attention and executive function predicted HRQOL within models that included ESS (sleepiness), SCPBI-Y (pain burden), and ED visits within the past year. The variance-inflation factors (VIF) in regression analyses were within the acceptable range (1.14 to 1.62), indicating that correlations between independent predictors are not inflating the regression coefficient due to multicollinearity. Our regression analyses indicated that ESS (sleepiness) and ED visits within the past year were significant predictors of HRQOL in both models, including CPT-3 commission errors and the BRIEF. Scores on the SCPBI-Y (pain burden) approached significance (*p* = 0.06).

We found that CPT-3 commission errors did not significantly predict HRQOL (*p* = 0.84). However, the regression analysis including the BRIEF GEC was the most explanatory (overall model fit *R*^2^ = 0.66) and demonstrated that the BRIEF GEC significantly predicted HRQOL (*p* = 0.04). Effect sizes for all predictors were large (η^2^ = 0.16–0.32) (see Table 4). Based on our data, with an estimated mean T score for the BRIEF GEC of 51.50 in our sample, for every 0.74 *increase* in the BRIEF T-score, there was a corresponding 1-point *decrease* in HRQOL indicating that as executive dysfunction increased, HRQOL decreased for paediatric patients with SCD.

**Table 4:** Two regression model with health-related quality of life (PedsQL) as the dependent variable and sleepiness (ESS), pain burden (SCPBI-Y), ED visits within the past year, with either executive function (BRIEF GEC) or CPT commission errors as predictors.

**Effect**	***B***	***B* 95% CI [LL, UL]**	**β**	***SE***	***r***	***Partial*** **η** ^**2**^	***p***	**Fit**
(Intercept)	134.46	[100.05, 168.87]						
Epworth Sleepiness Scale	−2.93	[−4.86, −0.99]	−0.46	0.90	−0.57	0.32	**0.005**	
Sickle Cell Pain Burden	−11.74	[−24.00, 0.53]	−0.29	1.61	−0.35	0.16	0.06	
Number of ED visits in the past year	34.41	[13.54, 55.29]	0.49	5.72	0.23	0.36	**0.003**	
BRIEF Global Executive Composite	−0.74	[−1.46, −0.02]	−0.33	0.33	−0.48	0.18	**0.04**	
								*R^2^* **=** **0.633**
								95% CI [0.25, 0.73]
(Intercept)	102.71	[60.01, 145.40]						
Epworth Sleepiness Scale	−3.44	[−5.48, −1.40]	−0.55	0.98	−0.58	0.36	**0.002**	
Sickle Cell Pain Burden	−14.04	[−27.27, −0.81]	−0.35	6.36	−0.37	0.18	**0.03**	
Number of ED visits in the past year	30.04	[2.06, 58.02]	0.42	13.45	0.23	0.19	**0.03**	
CPT-3 Commission Errors	−0.07	[−0.98, 0.85]	−0.03	0.44	−0.33	0.01	0.88	
								*R^2^* **=** **0.552**
								95% CI [0.14, 0.67]

*B represents unstandardised regression weights. LL and UL indicate the lower and upper limits of a confidence interval, respectively. β, standardised regression weights; SE, Standard Error; r, the zero-order correlation; Partial η^2^, partial eta squared. Significant differences are highlighted in bold (p < 0.05). ED, Emergency department; BRIEF GEC, Behavioural Rating Inventory of Executive Function Global Executive Composite; CPT-3, Conners' Continuous Performance Test-Third Edition*.

## Discussion

Our *post hoc* analysis of data from the POMS2b RCT demonstrated sleepiness, pain burden, ED visits, and executive dysfunction predicted HRQOL. All effect sizes were large and clinically significant. Although impulsivity was negatively correlated with HRQOL (*r* = −0.33), it was not a predictor within regression models. Demographic factors (i.e., age, gender, deprivation) and treatment variables (i.e., hydroxyurea use) did not independently predict HRQOL and laboratory values (i.e., haemoglobin, haematocrit, mean oxygen saturation) were not significantly correlated with HRQOL.

Patients with SCD often experience marked inequities influenced by geographic region, population density, socioeconomic status, care and pain outcomes, and disease management, along with reduced access and use of comprehensive specialist clinics (Mensah et al., 2019). Our findings did not show an association between these factors and HRQOL, but our patients were from a single geographical region and attended a comprehensive SCD clinic, which may explain why these factors had less of an influence on HRQOL. These sociocultural factors *should* be incorporated into the design, development, and implementation of skills-based programmes to ensure equitability and access. Within the context of pain coping interventions, however, the symptom targets that predicted HRQOL in the present study are defined, measurable, and most importantly, amenable to change.

Pain is often referred to as the “hallmark of SCD,” given that painful episodes and persistent pain frequently occur throughout the lifespan and are often the first presentation of the disease (Dampier et al., 2017). Although this focus is warranted, other pain coping targets (i.e., sleep) also play a role in HRQOL. Our data revealed the strong relationship between sleepiness and HRQOL and suggest incorporating measures to explore coping in relation to sleep difficulties (e.g., sleep-disordered breathing) and sleep hygiene behaviours (e.g., bedtime routine) may be a valuable addition to coping interventions for children with SCD. Our findings add to a growing body of literature that has demonstrated that more sleep disturbances (i.e., parasomnias and movement at night) were related to greater daytime sleepiness (Kölbel et al., 2020) and that there is a bi-directional relationship between poor night-time sleep quality and higher daytime pain in youth with SCD (Valrie et al., 2020). Expanding upon the influence of sleep, fatigue (a feeling of tiredness or exhaustion) has also been shown to be more strongly related to cognitive deficits in children with SCD (Rogers et al., 2017). In a non-SCD adult sample of patients with fibromyalgia, more sleep disturbances were associated with a reduced use of pain coping strategies (Theadom et al., 2007). Although further research is necessary with a paediatric SCD sample, sleepiness, cognitive function, and HRQOL are probably overlapping aspects of daytime function that may reduce the ability to cope with demands throughout the day, limiting the ability to use pain coping resources.

Although the number of ED visits within the past year was low in our sample, we did show that attending the ED had a large negative influence on HRQOL. This finding aligns with previous research in adults with SCD, where high ED utilisers experienced more distress and poorer HRQOL (Aisiku et al., 2009). Increased ED visits likely reflect increased pain, but increased ED visits also have an independent effect on HRQOL. Coping with the challenges of frequent hospital contacts and ED visits can be burdensome for patients with SCD. Being a disease that impacts a predominately Black population, SCD places patients at increased risk of racialised disparities. Patients with SCD report that race affects the quality of care and interpersonal relationships with hospital staff (Nelson and Hackman, 2013). Despite these significant barriers, a coping skills training programme has been shown to reduce ED visits in paediatric patients with SCD (Broome et al., 2001).

In non-SCD paediatric pain populations, multimodal interventions are effective for chronic pain management. They have demonstrated improvements in pain intensity, functional disability, anxiety, depression, catastrophising, school attendance, school functioning, and pain acceptance, with similar effectiveness seen whether the interventions were delivered in inpatient or outpatient settings. Successful non-pharmacological components of these interventions include group-based cognitive behavioural therapy (CBT), acceptance and commitment therapy (ACT), biofeedback, and acupuncture (Liossi et al., 2019). A wide diversity of non-pharmacological interventions have been assessed in adult and paediatric populations with SCD, including many of the successful components described. Other interventions that have also been tested include complementary therapies (e.g., prayer, guided imagery, music therapy) and physically-based therapies (e.g., massage therapy, yoga, aquatic rehabilitation) (Williams and Tanabe, 2016), as well adjuvant technology (e.g., virtual reality, smartphone app), which have been shown to improve self-efficacy and self-management about successfully managing SCD-related complications (Agrawal et al., 2019; Crosby et al., 2020, 2021; Hood et al., 2020a). However, most of these interventions are not multimodal, have primarily assessed acceptability and feasibility, and have been conducted with small samples at a single institution (Williams et al., 2018). Future interventions will need to be on a larger scale, include randomisation, and incorporate a biopsychosocial approach with significant patient involvement to increase their engagement in improving pain-coping resources and HRQOL.

In light of the fact that executive function difficulties have frequently been demonstrated for patients with SCD (Hood et al., 2019, 2020b; Prussien et al., 2019) and that executive function was predictive of poorer HRQOL in this study, we would suggest the utilisation of multimodal interventions that include components of executive function training or monitoring. Generally, studies assessing how to improve executive function in patients with SCD have not incorporated pain assessment, coping, or management (Marshall et al., 2009; Hood et al., 2020b). However, one small pilot study did demonstrate that participants who completed more of the executive function training programme had lower pain impact scores on the PedsQL, providing a promising avenue for future research (Hardy et al., 2016).

Across the symptom categories identified here, adequate cognitive function is required to process and work through any potential hurdles. Patients with SCD experience specific challenges in the cognitive domains that, if improved, would make managing pain and their treatment regimen easier. Cognitive deficits in processing speed, attention, and executive function make it more difficult to quickly process information from medical providers and maintain sustained attention over long periods. Further, the integration of data from multiple sources along with balancing needs and priorities also requires the ability to process higher-order information. Our study found that executive dysfunction measured through caregiver-report was related to HRQOL, but executive, attention, and processing speed test scores were not associated. Difficulties in completing everyday behaviours in the school and home environment (e.g., time management, organisation) may have a greater effect on HRQOL, emphasising the need for future studies to incorporate ecologically valid measures (Berg et al., 2012). Adaptations to pain coping interventions to account for and better accommodate these cognitive challenges could include clear communication that can be understood the first time it is read or heard. Using plain or jargon-free language is not “dumbing things down”; instead, it ensures information is accessible for the intended audience. Other modifications could include integrating frequent and immediate feedback during components of pain coping skills training, shorter, more frequent sessions, and breaking down tasks and materials (e.g., handouts) into small, manageable steps.

Some limitations influence the present study's findings that should be addressed. Although a strength of research was that our sample was drawn from an RCT, and pain burden was measured each month over 8 months, our data primarily focuses on baseline assessments. More robust and longitudinal research designs are needed to thoroughly appreciate the consequences of sleepiness, pain burden, ED utilisation, and executive dysfunction on paediatric patients with SCD. It is also possible that including pain burden scores collected after assessing HRQOL could have influenced results. Additionally, our sample size was small, so we did not have the power to detect smaller effects, and possible interactions between variables (e.g., pain burden and sleep) and analyses of specific PedsQL subscales could not be assessed. Replication in a larger sample would provide greater clarity. This study was conducted at large, urban institutions in the UK, and the cohort may not be representative of the paediatric patients internationally. Although we collected data to assess hydroxyurea use, adherence to this treatment was not included in our study protocol. Our study did not include a direct measure of pain coping, which would have allowed us to test the relationship with our variables of interest. However, not having a specific pain coping measure allowed us to consider whether more generic coping scales that assess a range of outcomes might be more useful, given the complexity of the challenges faced by patients with SCD.

Our study aimed to elucidate the symptom categories influencing HRQOL to determine targets for future multimodal pain coping and HRQOL interventions. We found that demographic and medical variables were weakly related to HRQOL; whereas, sleepiness, pain burden, ED visits, impulsivity, and executive dysfunction were more strongly related to HRQOL. Notably, almost all of the strongly correlated variables (except impulsivity) independently predicted HRQOL. Although thoughtfulness about sociocultural factors is critically needed to develop programmes and retain patients with SCD in research and clinical practise, we have shown that specific symptom-based targets for pain coping are measurable and may be responsive to change through intervention. As we consider helping to improve the lives of this paediatric population, it is necessary to expand beyond the measurement of pain intensity and instead integrate the assessment of measurable symptom categories that may serve as targets to improve HRQOL that are responsive to change.

## Data Availability Statement

The datasets presented in this article are not readily available because the datasets generated and analysed during the current study are available from the corresponding author on reasonable request and in accordance with ethical restrictions imposed by the Ethics Committees that approved this study. Requests to access the datasets should be directed to fenella.kirkham@ucl.ac.uk.

## Ethics Statement

The study received ethical approval from the NRES Committee East of England – Cambridge South (14/EE/0163). Written informed consent to participate in this study was provided by the participants' legal guardian/next of kin.

## Author Contributions

FK is the chief investigator, she conceived the study, grant application, and protocol development. CL made significant contributions to the design and implementation of the study. AH drafted this work and managed all revisions. AH, HS, MK, JK, and AS contributed to the study design and development of the proposal. BI, MP, JH, SC, SH, and MA made contributions to the conception of the study, the design of the work, and data acquisition. All authors read and approved the final manuscript.

## Funding

The National Institute for Health Research (UK; PB-PG-1112-29099) provided funding for patient recruitment. AH was supported by a grant from the National Heart, Lung, and Blood Institute, National Institutes of Health (1F32HL143915). MK and HS were funded by Action Medical Research (GN2509) and JK by Great Ormond Street Children's Charity (V4615).

## Conflict of Interest

FK has received honoraria from Novartis and Bluebird Bio and has served as a consultant for Global Blood Therapeutics. AS is currently employed by Axio Research LLC and has served as a consultant for Providence Medical Technology, Intuitive Surgical Inc., Laborie, Janssen Pharmaceuticals, Pulse Biosciences, Stryker Corporation. The remaining authors declare that the research was conducted in the absence of any commercial or financial relationships that could be construed as a potential conflict of interest.

## Publisher's Note

All claims expressed in this article are solely those of the authors and do not necessarily represent those of their affiliated organizations, or those of the publisher, the editors and the reviewers. Any product that may be evaluated in this article, or claim that may be made by its manufacturer, is not guaranteed or endorsed by the publisher.
